# Impact on public attitudes of a mental health audio tour of the National Gallery in London

**DOI:** 10.1111/eip.13268

**Published:** 2022-01-31

**Authors:** Simon Riches, Natalie Steer, Ruxandra Vasile, Sophie Lyles, Laoise O'Reilly, Martina Guiotto, Tom Hughes, Meagan McKay, Megan Westhead, Rachel M. Latham, Joanne B. Newbury, Anna Murray, Amber Goneni, Aleksandra Orehova, Rachel Temple, Rose Thompson, Fiona Houston, Helen L. Fisher

**Affiliations:** ^1^ King's College London, Social, Genetic & Developmental Psychiatry Centre Institute of Psychiatry, Psychology & Neuroscience London UK; ^2^ King's College London, Department of Psychology Institute of Psychiatry, Psychology & Neuroscience London UK; ^3^ South London and Maudsley NHS Foundation Trust Bethlem Royal Hospital Kent UK; ^4^ King's College London, Department of Psychosis Studies Institute of Psychiatry, Psychology & Neuroscience London UK; ^5^ Sussex Partnership NHS Foundation Trust Worthing UK; ^6^ Faculty of Brain Sciences, Division of Psychology and Language Sciences University College London London UK; ^7^ ESRC Centre for Society and Mental Health King's College London London UK; ^8^ Bristol Medical School: Population Health Sciences University of Bristol Bristol UK; ^9^ The National Gallery London UK; ^10^ The McPin Foundation London UK; ^11^ Antenna International London UK

**Keywords:** apps, art, experiential learning, lived experience, stigma

## Abstract

**Aim:**

The arts have the potential to increase public awareness about mental health and reduce stigma. However, arts‐based projects to raise awareness have been small‐scale. In this study, a mental health‐awareness audio tour of The National Gallery in London was co‐produced and narrated by young adults with relevant lived experience. The study investigated the acceptability of the tour to the public and evaluated its impact on public attitudes about mental health.

**Methods:**

Participants were Gallery visitors over four consecutive days. The tour led visitors on 10 stops through the Gallery. Each stop focused on artworks and Gallery spaces, challenged common myths about mental health, and invited visitors to consider their personal views. Participants completed measures of mood and attitudes about mental health pre‐ and post‐tour and provided narrative feedback.

**Results:**

Pre‐tour, participants (*N* = 213) reported high levels of happiness, compassion towards people with mental health conditions, comfort talking about mental health, and positive attitudes about mental health. Post‐tour, participants (*N* = 111) reported significant increases in happiness, comfort, and positive attitudes. In feedback, participants (*N* = 85) reported that strengths of the tour were the music, inclusion of lived experience, art and mental health links, and reported that the tour was informative, innovative, and improved mental health awareness.

**Conclusions:**

The tour increased positive attitudes, despite positive baseline attitudes, indicating the feasibility of arts‐based interventions in major venues to reduce stigma. Sampling limitations and participant retention suggest that arts‐based projects to raise awareness should target more diverse audiences and consider data collection strategies in large venues.

## 1 INTRODUCTION

Mental health stigma is characterized by discriminatory behaviours, prejudicial attitudes, and biased social structures towards those with mental health conditions (Corrigan, 2000). Misconceptions and negative attitudes about mental health conditions are highly prevalent (Kaur et al., 2016), which can have wide‐ranging consequences for individuals experiencing these conditions, often considered to be worse than the condition itself (Thornicroft et al., 2016). Among individuals with mental health conditions, public stigma is associated with social disadvantages and negative outcomes, representing a major health inequality (Pedersen & Paves, 2014; Clement et al., 2011). The World Health Organization have prioritized reductions in mental health stigma and discrimination (World Health Organization, 2013). However, despite anti‐stigma campaigns, such as the UK *Time to Change* campaign, mental health stigma remains a significant issue (Evans‐Lacko et al., 2014) and further interventions are necessary. Meta‐analysis evidence suggests direct, face‐to‐face campaigns are the most impactful anti‐stigma approach for meaningful behaviour change (Corrigan et al., 2012).

The arts have the potential to increase public awareness about mental health and reduce stigma (Gronholm et al., 2017) by inviting reflection, discussion, and eliciting empathy (Ho et al., 2017). Galleries are enhancing public engagement by using technologies to create more interactive, immersive experiences (Falco & Vassos, 2017; Riches, Maskey, Waddingham, et al., 2018) and co‐producing art projects with people with lived experience for a more authentic and personal experience (Riches, Maskey, Dishman et al., 2018; Ho et al., 2017). Despite these advances, projects have been small‐scale and generally outside mainstream settings.

The present study evaluated a mental health‐awareness audio tour of The National Gallery in London. The tour was co‐produced with young adults with lived experience of mental health issues through workshops based in the Gallery (Riches et al., n.d.). The aim of this study was to investigate the acceptability of the tour for Gallery visitors and evaluate its impact on public attitudes about mental health. Hypotheses were that participants would find the tour acceptable, and that it would increase understanding and improve attitudes about mental health.

## 2 METHODS

### 2.1 Design, participants and setting

A cross‐sectional within‐subjects design was used to evaluate the impact of the mental health audio tour on public attitudes about mental health. The National Gallery is a large, free‐admission art gallery in central London; and is one of the most visited galleries in the world (www.nationalgallery.org.uk). Participants were visitors to the National Gallery from the general public who undertook the audio tour. This project was an audit of the audio tour conducted with the approval of the National Gallery; therefore, no ethical approval was obtained. However, the study was conducted in accordance with The Declaration of Helsinki (1964).

### 2.2 National Gallery Mental Health audio tour

This project was a collaboration between staff and Young Producers from the National Gallery, young people with lived experience of mental health issues, technologists who produce audio guides, and academics from King's College London. The aim of the project was to develop an audio tour of the National Gallery that raised awareness and debunked myths about mental health by promoting the voices of young adults with lived experience of mental health issues. Development stages of the tour included workshops in the Gallery with young adults with lived experience of mental health issues, writing the audio tour script, creation of a progressive web app that could be accessed by mobile devices, and recording the tour narration. Further details of the development of the tour are provided in Riches et al. (under review). The tour was launched on 10th October 2019 and was free to all Gallery visitors for 6 months in the first instance. The tour comprised 10 stops, each focusing on artworks or Gallery spaces, identified and challenged common myths about mental health, and invited listeners to consider their personal views and reflect on their wellbeing. The tour was narrated by two young adults who were part of the National Gallery's Young Producer's programme and included excerpts of four young adults with relevant lived experience reflecting on their own personal experiences and the artworks. See Table 1 for a description of all stops, artworks, and myths.

**TABLE 1:** Summary of the stops included in the National Gallery mental health audio tour

Audio stop and myth	Artworks/gallery space	Link between myth and artworks/gallery space
1. Mental health issues are a modern phenomenon—young people are too sensitive nowadays.	The Sainsbury Wing of the Gallery, room 51.	This is the newest wing of the Gallery but houses the oldest paintings thus mirroring how mental health issues have been around for centuries even though they may appear to be a modern phenomenon because people are more comfortable talking about them now.
2. Everyone experiences mental health issues in the same way.	Two paintings of ‘The Virgin & Child’ by Giovanni Battista Cima da Conegliano, room 57.	The two paintings depict the same subject but from different perspectives. In the same way, experiences of the same mental health issues are often different and vary between people.
3. Hearing voices and seeing things are rare and a sign of mental illness.	‘The Vision of the Blessed Gabriele’ by Carlo Crivelli, room 57.	Historically, having visions and hearing voices, such as depicted in this painting, were experiences that were celebrated and indicated that someone was special rather than unwell. Many people in the general population without mental illnesses have these experiences.
4. People are destined to develop mental health issues from birth.	Landscape paintings, room 19.	Listeners heard two contrasting pieces of music to see how it changed their emotional reaction to the paintings. Similarly, our experiences impact our emotional wellbeing and although our genes may increase the likelihood of mental health conditions, they are not deterministic.
5. Ethnic minorities or those from developing countries are not affected by mental health issues.	‘The Adoration of the Kings’ by Jan Gossaert, room 14.	This is one of the very few paintings depicting people of colour in the Gallery. In the same way that ethnic minorities initially seem ‘missing’ from classical art, mental health issues can also seem less visible among those from ethnic minorities or low‐income countries because of a reluctance to speak out due to fear of stigmatization or institutional barriers to accessing care.
6. People with mental health issues are lazy, attention seeking and manipulative.	Boris Anrep Mosaics, in the Portico, main entrance of the Gallery.	The Mosaics include depictions of Virginia Woolf and Winston Churchill, who both achieved great things while battling their mental health issues.
7. An artist needs to have mental health issues in order to be creative.	Paintings by Van Gogh, Henri de Toulouse‐Lautrec, and Claude Monet, Impressionists Gallery, room 43.	Although some artists, such as those whose paintings are featured in this room, have experienced mental health issues, they had periods when they were too unwell to paint. The ‘tortured artist’ stereotype can be damaging as it may prevent or delay people in creative jobs from seeking the help that they need, which may make their mental health issues much worse.
8. Once you have a mental health issue, it will affect you for the rest of your life.	‘An Experiment on a Bird in the Air Pump’ by Joseph Wright ‘of Derby’, room 34.	Like the uncertainty of the outcome of the bird in the painting, the outcome of mental illness is uncertain. Many make a full recovery; others have periods where they feel well.
9. Mental health issues are a sign of weakness.	The Barry Rooms, room 36.	During the second World War the pianist Myra Hess performed concerts in this room even during the Blitz to demonstrate London's resilience. People with mental health issues show similar strength and determination when dealing with these often very difficult experiences every day.
10. People with mental health issues are unpredictable, dangerous, and violent.	‘An English Vessel and a Man‐of‐war in a Rough Sea’ by Ludolf Bakhuizen, Gallery A.	This painting depicts a ship attempting to cross a turbulent sea with the sky overhead threatening to unleash a storm at any minute reflecting the common myth that people with mental health issues are unpredictable and may be dangerous or violent. Whereas they are actually more likely to experience violence from others and hurt themselves.

### 2.3 Procedure

The tour was promoted on the Gallery website and social media. Promotion described a free mental health‐themed audio tour exploring art and mental health myths. The evaluation was conducted on the first four consecutive days after the tour was launched (i.e., 10th–13th October 2019). On entry to the Gallery, visitors on these days were approached by a researcher and invited to undertake the mental health audio tour and complete a brief survey before and after the tour. Researchers used tablets to administer surveys that were designed on the online software program Qualtrics. Following completion of the pre‐tour survey, participants accessed the tour by connecting to the free Gallery Wi‐Fi and using the web‐based app (https://tour.aiwebservices.com/c/ngmentalhealth) on their mobile phone. They wore headphones to listen to the audio. Researchers checked that they were able to access the tour and assisted with technical issues. Participants were advised to listen to the Introduction audio for the tour first before setting off to explore the Gallery. They were provided with a map of the Gallery indicating the relevant room for each stop and were free to choose the order in which they visited the stops. Participants were told by researchers that they could remove their headphones at any time if they felt uncomfortable. A clinical psychologist was always present in the Gallery to monitor visitors for distress and address concerns. Participants were asked to find researchers to complete the post‐tour survey once they had finished listening to all stops. Researchers were positioned near the main entrances and exits to maximize the likelihood of engaging with visitors as they entered the Gallery to invite them to take part and also to prompt participants leaving the Gallery to complete the post‐tour survey. Participant anonymity was ensured by using a unique four‐digit participant ID number for data collection which was provided on a sticker that participants were asked to affix to their clothing. This meant that the same number could be entered by a researcher into the tablet for the post‐tour survey to ensure it could be linked with their pre‐tour survey responses. A prize draw for a £50 shopping voucher incentivized participation each day.

### 2.4 Measures

Pre‐tour, participants self‐reported their demographic characteristics of age, gender and ethnicity, and completed 11‐point visual analogue scales (VAS), from 0 (‘Not at all’) to 10 (‘Very much’), on current happiness, stress, compassion towards people with mental health conditions and comfort talking to people about mental health, which were adapted from previous arts and mental health research (Riches, Maskey, Dishman, et al., 2018; Riches, Maskey, Waddingham, et al., 2018). Participants then completed 18 items, from 1 (‘strongly disagree’) to 5 (‘strongly agree’), of the 26‐item Attitudes to Mental Illness scale (Time to Change, 2014). Items for inclusion were selected according to appropriateness for the setting. The scale has four subscales: eight items were selected from the ‘Fear and exclusion of people with mental illness’ subscale, three from ‘Understanding and tolerance of mental illness’ subscale, six from ‘Integrating people with mental illness into the community’ subscale, and one from ‘Causes of mental illness and the need for special services’. Item wordings received minor adaptations to maximize neutrality, inclusivity, and to correspond to contemporary usage, for example, ‘mental patients’ and ‘the mentally ill’ were adapted to ‘people with mental health conditions’ throughout, and the original item ‘A woman would be foolish to marry a man who has suffered from mental illness’ was adapted to ‘People would be foolish to begin a relationship with someone who has had a mental health condition’. See Table 2 for all pre‐tour items.

**TABLE 2:** Pre‐ and post‐tour mood and attitudes towards mental health conditions

	Baseline (*N* = 213)	Pre‐ and post‐tour comparison (*N* = 111)				
Item	Pre‐tour	Pre‐tour	Post‐tour	Test statistic	*p*	Effect size (*d*)
*Visual analogue scales: ‘*Please mark on the line to indicate…’	M (SD)	M (SD)	M (SD)			
…how happy you feel right now	7.54 (1.7)	7.54 (1.5)	7.96 (1.5)	−3.31	0.001*	0.28
…how stressed you feel right now	3.59 (2.5)	3.46 (2.3)	2.89 (2.3)	2.32	0.006*	0.25
…how compassionate you feel towards people with mental health conditions	8.37 (1.7)	8.48 (1.5)	8.67 (1.5)	−1.81	0.073	0.13
…how comfortable you would be talking to a friend, colleague, or family member about their experience of having a mental health condition	8.09 (2.0)	7.99 (2.1)	8.42 (1.7)	−2.66	0.009*	0.23
…how you feel your attitude towards people with a mental health condition has changed as a result of this tour	–	–	6.69 (2.4)	–	–	–
…how much you enjoyed the tour	–	–	7.29 (2.3)	–	–	
…how much you learned about mental health from the tour	–	–	5.61 (2.6)	–	–	
…the extent to which the tour made you think differently about your own mental wellbeing	–	–	5.23 (2.9)	–	–	
…the extent to which the tour provided information that you could understand	–	–	8.00 (2.3)	–	–	
*Attitudes to Mental Illness items* (agreement with item)	*N* (%)	*N* (%)	*N* (%)			
Locating mental health facilities in a residential area downgrades the neighbourhood	24 (11.3)	8 (7.3)	4 (3.7)	–	0.727	0.07
It is frightening to think of people with mental health conditions living in residential neighbourhoods	16 (7.5)	4 (3.7)	3 (2.8)	–	0.625	0.09
I would not want to live next door to someone who has a mental health condition	17 (8.0)	6 (5.5)	4 (3.7)	–	0.500	1.13
People would be foolish to begin a relationship with someone who has had a mental health condition, even though they seem fully recovered	9 (4.2)	1 (0.9)	4 (3.7)	–	0.250	0.22
Anyone with a history of a mental health condition should be excluded from taking public office	12 (5.6)	3 (2.8)	4 (3.7)	–	1.000	0
People with a mental health condition should not be given any responsibility	9 (4.2)	4 (3.7)	3 (2.8)	–	1.000	0
People with a mental health condition are a burden on society	7 (3.3)	1 (0.9)	3 (2.8)	–	0.625	0.09
As soon as a person shows signs of a mental health condition, they should be hospitalized	9 (4.2)	4 (3.6)	5 (4.6)	–	1.000	0
Virtually anyone can develop a mental health condition	185 (86.9)	95 (87.2)	98 (89.9)	–	1.000	0
People with a mental health condition do not deserve our sympathy	4 (1.9)	2 (1.8)	3 (2.8)	–	1.000	0
We need to adopt a far more tolerant attitude towards people with a mental health condition in our society	197 (92.5)	101 (92.7)	105 (96.3)	–	1.000	0
People with mental health conditions are far less of a danger than most people suppose	150 (70.4)	83 (76.1)	91 (83.5)	–	0.143	0.28
Less emphasis should be placed on protecting the public from people with mental health conditions	103 (48.4)	60 (55.0)	74 (67.9)	–	0.035*	0.41
Residents have nothing to fear from people coming into their neighbourhood to obtain mental health services	172 (80.8)	92 (84.4)	94 (86.2)	–	1.000	0
People with mental health conditions should have the same rights to a job as anyone else	190 (89.2)	102 (93.6)	105 (96.3)	–	0.625	0.09
Mental illness is an illness like any other	162 (76.1)	80 (73.4)	90 (82.6)	–	0.021*	0.45
No‐one has the right to exclude people with mental health conditions from their neighbourhood	185 (86.9)	99 (90.8)	105 (96.3)	–	0.125	0.30
There is something about people with mental health conditions that makes it easy to tell them apart from ‘normal’ people	32 (15.0)	12 (11.0)	8 (7.3)	–	0.607	0.10
*Attitudes to Mental Illness survey subscales*	*Mdn* (Ra)	*Mdn* (Ra)	*Mdn* (Ra)			
Fear and exclusion of people with mental illness	10 (40)	10 (25)	9 (29)	−3.002	0.003*	0.60
Understanding and tolerance of mental illness	15 (15)	15 (15)	15 (10)	−1.266	0.205	0.24
Integrating people with mental illness into the community	26 (30)	26 (30)	28 (24)	−3.317	0.001*	0.67
Causes of mental illness and the need for special services	1 (5)	1 (5)	1 (5)	−1.017	0.309	0.20

*Note*: Test statistic = Paired‐Samples T‐Test for Visual analogue scales, McNemar's Test for Attitudes to Mental Illness survey items, Wilcoxon Signed‐Rank Test for Attitudes to Mental Illness survey subscales. Effect sizes were calculated using Cohen's *d*. Attitudes to Mental Illness scores were dichotomised into agree or disagree, table shows the ‘agree’ percentages. *N* = 2 missing data in post‐tour Attitude to Mental Illness survey items and subscales.

Abbreviations: M, mean, Mdn, median. Ra, range; SD, standard deviation.

Post‐tour, participants were invited to recomplete all pre‐tour VAS, the Attitudes to Mental Illness items and five additional VAS on attitude change, enjoyment, amount learned, the extent to which they thought differently about their own mental wellbeing, and how understandable they found the tour. See Table 2 for all post‐tour items. Participants were also invited to provide post‐tour narrative feedback, verbally or in writing, in response to researcher questions, which included ‘Can you tell us about your experience of the mental health tour? Which parts of the tour had the most impact on you? What else could we include? Any other comments about the mental health tour?’

### 2.5 Analysis

Quantitative data were analysed using SPSS v26. At baseline, mean pre‐tour survey VAS scores were calculated for each item. To evaluate agreement with items of the Attitudes to Mental Illness survey, total percentage of agreement for each item was calculated by dichotomising each item into ‘agree’ (scores of 4 and 5, recoded to =1) or ‘disagree’ (scores of 1, 2 and 3, recoded to =0). Total subscale scores were calculated by summing original scores (coded 1–5) for each subscale. Note, one item (‘People with a mental health condition don't deserve our sympathy’) was reverse scored prior to deriving the relevant subscale to ensure all items were coded in the same direction before summing them. High mean scores from items in ‘Fear and exclusion of mental illness’ and ‘Causes of mental illness and the need for special services’ subscales indicate more negative attitudes. High mean scores from items in ‘Understanding and tolerance of mental illness’ and ‘Integrating people with mental illness into the community’ subscales indicate more positive attitudes. To explore baseline scores for Attitudes to Mental Illness subscales, original scores (coded as 1–5) for each sub‐section were summed, and median and range was calculated as data violated parametric assumptions. Pre‐ and post‐tour mood and attitudes were compared to evaluate acceptability of the tour and attitude change. To explore potential confounding variables between pre‐ and post‐tour survey participants, Chi Square analysis examined if they significantly differed in age (grouped by ≤35 or ≥36), gender, and ethnicity (grouped by ‘White British’ vs. all other groups). A paired‐samples *t*‐test compared pre‐ and post‐tour mean VAS for participants with data available at both time‐points. A McNemar Test assessed pre‐ and post‐tour change in dichotomised agreement scores with Attitudes to Mental Illness survey items. A Wilcoxon matched‐pairs signed‐rank test compared pre‐ and post‐tour median Attitudes to Mental Illness subscale scores as data violated parametric assumptions. Effect sizes were calculated using Cohen's *d*. Verbal feedback was anonymised and transcribed. Written and verbal feedback were pooled. Qualitative data were analysed thematically using NVivo v12. ID numbers were not recorded for participants who gave verbal feedback. Themes were organized by researchers into categories of *strengths* and *weaknesses* of the tour.

## 3 RESULTS

Two‐hundred and thirteen participants took the tour and completed the pre‐tour survey. Table 3 reports their demographic characteristics. One‐hundred and twenty‐nine (60.6%) were female, 145 (68.1%) were ≤35 years of age, and 64 (30.0%) were of White British ethnicity. Table 2 reports pre‐tour mood and attitudes. Mean VAS scores indicate high levels of happiness, compassion towards people with mental health conditions, and comfort talking about mental health, and low stress. Attitudes to Mental Illness item scores showed a higher percentage of agreement with positively worded items and lower percentage of agreement with negatively worded items, indicating positive baseline attitudes towards those with mental health conditions. Subscale scores were highest for ‘Integrating People with Mental Illness into the Community’ and lowest for ‘Causes of Mental Illness and The Need for Special Services’, indicating positive attitudes prior to undertaking the tour.

**TABLE 3:** Participants demographic characteristics for pre‐tour survey (*N* = 213), post‐tour survey (*N* = 111) and post‐tour narrative feedback (*N* = 85)

Demographics	Pre‐tour *N* (%)	Post‐tour *N* (%)	*X* ^*2*^ (*df*)	*p*	Feedback *N (%)*
Age					
Under 18	3 (1.4)	0 (0)	–	–	0 (0)
18–25	82 (38.5)	43 (38.7)	–	–	34 (40.0)
26–35	63 (29.6)	32 (28.8)	–	–	22 (25.9)
36–45	30 (14.1)	17 (15.3)	–	–	14 (16.5)
46–55	15 (7.0)	8 (7.2)	–	–	6 (7.1)
56–65	9 (4.2)	4 (3.6)	–	–	4 (4.7)
Over 65	8 (3.8)	5 (4.5)	–	–	4 (4.7)
Preferred not to say	3 (1.4)	2 (1.8)			1 (1.2)
Age groupings					
≤35 years	145 (68.1)	75 (67.6)	–	–	56 (65.9)
≥36 years	62 (29.1)	34 (30.6)	0.05 (1)	0.820	28 (32.9)
Gender					
Female	129 (60.6)	69 (62.2)	–	–	54 (63.5)
Male	80 (37.6)	38 (34.2)	0.23 (1)	0.631	28 (32.9)
Other	0 (0)	0 (0)			0 (0)
Preferred not to say	4 (1.9)	4 (3.6)			3 (3.5)
Ethnicity					
Asian/Asian British	15 (7.0)	7 (6.3)	–	–	6 (7.1)
Black/Black British	6 (2.8)	3 (2.7)	–	–	4 (4.7)
White British	64 (30.0)	42 (37.8)	–	–	31 (36.5)
White Other	100 (46.9)	46 (41.4)	–	–	34 (40.0)
Mixed/Multiple	16 (7.5)	7 (6.3)	–	–	6 (7.1)
Other	7 (3.3)	2 (1.8)			2 (2.4)
Preferred not to say	5 (2.3)	4 (3.6)			2 (2.4)
Ethnic groupings					
White British	64 (30.0)	42 (37.8)	–	–	31 (36.5)
All other ethnic groups	137 (64.3)	63 (56.8)	2.03 (1)	0.154	50 (58.8)

*Note*: ‘Under 18’, ‘Other’ and ‘Preferred not to say’ options were excluded from Chi Square Tests.

Abbreviations: df, degrees of freedom; *X* ^*2*^, Chi Square test.

One‐hundred and eleven pre‐tour survey participants (52.1%) returned to complete the post‐tour survey. Table 3 reports their demographic characteristics. Sixty‐nine (62.2%) were female, 75 (67.6%) were ≤35 years of age, and 42 (37.8%) were of White British ethnicity. Mean differences in pre‐ and post‐tour participants' age, gender and ethnicity were not significant (see Table 3). Table 2 reports pre‐ and post‐tour mood and attitudes. There were significant increases in happiness and comfort, and decreases in stress, all with small effects. Compassion increased but it was not statistically significant. Post‐tour mean VAS scores were high for enjoyment and how understandable participants found the tour; they were moderate for attitude change, amount learned, and the extent to which participants thought differently about their own mental wellbeing. Post‐tour, changes in agreement with the Attitudes to Mental Illness items ‘Less emphasis should be placed on protecting the public from people with mental health conditions’ and ‘Mental illness is an illness like any other’ were statistically significant, with small‐moderate effects. Changes for all other items were not significant. The Attitudes to Mental Illness subscales, ‘Fear and Exclusion of People with Mental Illness’ and ‘Integrating People with Mental Illness into the Community’, were significantly more positive post‐tour, both with large effects. Changes in ‘Understanding and Tolerance of Mental Illness’, and ‘Causes of Mental Illness and the Need for Special Services’ subscales were not significant.

Eighty‐five of the 111 post‐tour participants (76.6%) provided narrative feedback. Fifty‐four (63.5%) were female, 56 (65.9%) were ≤35 years of age, and 65 (76.5%) were of White ethnicity. Table 4 reports themes, explanations, and illustrative quotes. Participants reported that strengths of the tour were the *integration with music*, inclusion of *lived experience*, the *art and mental health links*, and reported that the tour was *informative*, *innovative*, and *improved mental health awareness*. A weakness was the *tangential links between artworks and audio tour content* for some of the stops.

**TABLE 4:** Thematic analysis of participants' experiences of the tour (*N* = 85)

Theme	Explanation	*N* (%)	Illustrative quotes	
Strengths				
Integration with music	Participants enjoyed the music stop and felt that the music made them think.	22 (26)	‘Enjoyed the parts with the music a lot’. (#0351) ‘I really liked the landscape paintings with the two different types of music, had not really thought very deeply about how listening to music can affect your outlook on very simple daily things’. (No ID)	
Lived experience	Participants reported they felt moved by the young people's experiences and gained insight from them.	21 (25)	‘My favourite part…I guess the interviews the young people gave with lived experience, very good and quite moving as well’. (No ID) ‘Hearing about other people's experience of mental health was refreshing and insightful’. (#0221)	
Informative	Participants found the audio tour to be informative and appreciated gaining this information in the gallery setting.	21 (25)	‘It was really informative. The information was very relatable and gave a great insight into mental health through the ages as well as its link to the arts. I really enjoyed it!’ (#0265) ‘I really enjoyed the tour and the variety of information. It meant a lot to me that these experiences were being examined in such a popular space’. (#0417)	
Innovative	Participants thought the tour was an interesting way to explore the art and to raise awareness of mental health.	20 (24)	‘Very innovative and interesting approach to increase awareness’. (#0154) ‘Interesting way of exploring the art’. (#0258)	
Improves mental health awareness	Participants reported feeling that this audio tour is a good step towards better mental health awareness.	20 (24)	‘I feel offering this tour is a very positive step forwards and I hope people take the opportunity to take the tour’. (#0367)	
Art and mental health links	Participants felt that using art to raise awareness of mental health worked well and that the link between the two is interesting.	14 (17)	‘I think the tour is so valuable because it does help to raise awareness through art, which is a great medium to express ourselves and heal’. (#0160) ‘I think there's a very interesting link between mental health and art’. (No ID)	
Weaknesses				
Tangential links between artworks and audio tour content	Participants reported some tangential links between the content of the audio tour and the art.	27 (32)	‘I felt [the audio tour] could have benefitted from linking more closely with the paintings’. (#0230)

## 4 DISCUSSION

This study aimed to evaluate the impact on public attitudes of a mental health audio tour of the National Gallery in London. As hypothesised, participants found the tour acceptable, it increased understanding and led to more positive attitudes about mental health. High scores for enjoyment and how understandable participants found the tour indicate that participants had a positive and engaging experience and highlight the potential for future public engagement and anti‐stigma campaigns in large‐scale cultural spaces. Large effects for reducing negative attitudes about fear and exclusion of people with mental health conditions and enhancing positive attitudes on integration of such individuals into the community are comparable to findings from similar art‐based anti‐stigma interventions (Aldam et al., 2017; Kosyluk et al., 2018), and positive effects on happiness, stress, and comfort are consistent with other interactive arts‐based experiences (Riches, Maskey, Waddingham, et al., 2018). The experiential dimension to the tour and inclusion of the voices of people with lived experience enabled visitors to engage with complex topics and reflect on their social and personal meaning. This appears to be crucial to instigating attitude change given that experiential learning theory suggests active engagement is more valuable and effective than many other educational approaches (Kolb et al., 2001).

Strengths of the study included the novel arts‐based approach in a world‐renowned art gallery, co‐production with people with lived experience, and a relatively large sample from the general population. The study provides evidence in favour of using art galleries as spaces for raising awareness about mental health in creative and engaging ways. This has important public health benefits as better mental health awareness is linked to lower levels of stigma and discrimination against those experiencing psychological difficulties (Evans‐Lacko et al., 2014), which in turn may lead to those with mental health conditions seeking help earlier and potentially improving their prognosis (Clement et al., 2015; Schnyder et al., 2017).

Limitations include lack of diversity in the sample, use of some untested scales, and lack of a control group and longitudinal data to identify more sustained attitude or behaviour change. Demographic data indicates that the tour disproportionally reached young adults, people of White ethnicity and women, limiting generalisability of the findings. Baseline positive attitudes and comfort discussing mental health contrasted with that found by a meta‐analysis showing public attitudes towards mental health conditions are generally negative (Schomerus et al., 2012). However, the sample may not be representative of the general population, as it only included individuals who chose to attend a major art gallery. Although the arts appear to be a powerful medium to reduce stigma and stimulate attitude change, arts consumers have been found to have higher openness to experience, a trait associated with lower stigmatizing attitudes (Bachleda & Bennani, 2016; Fino et al., 2019). In addition, on‐the‐spot recruitment may indicate that participants had a prior interest in mental health, leading to higher baseline awareness of mental health issues. Therefore, non‐significance of changes in most Attitudes to Mental Illness items are likely to be due to the generally very positive attitudes at baseline.

Future research might include a larger, randomly selected sample with more diverse ethnic and age representation, in addition to a control group, standardized attitudes and stigma measures, and a longer‐term follow‐up to assess whether increases in positive attitudes are sustained. Research suggests art gallery visitors may be of higher socio‐economic status (Evrard & Krebs, 2018), so future arts‐based anti‐stigma campaigns or experiences could aim to reach audiences from a wider range of socio‐economic positions through mainstream cultural or entertainment media. Educational benefits of such awareness‐raising experiences may extend to specialist groups, such as students and healthcare professionals (Riches et al., 2019). The narrative post‐tour feedback in this study was brief due to the busy and crowded Gallery, which might explain the relatively low percentage endorsement for most common themes. Therefore, future studies might consider conducting longer follow‐up interviews with a small random selection of participants. Furthermore, participant retention rates between pre‐ and post‐tour surveys in this study gives an indication of the challenges involved in collecting research data in major national galleries, which has been reported by researchers involved in the tour (Riches et al, under review). Future arts‐based projects on this scale may need to consider alternative strategies for data collection.

In conclusion, this study indicates that a mental health‐themed audio tour of the National Gallery in London, which was co‐produced with young adults with lived experience, was a novel and effective way to raise public awareness about mental health and reduce stigmatizing attitudes. Future arts‐based anti‐stigma projects should aim to replicate this impact on broader and more diverse audiences.

## CONFLICT OF INTEREST

The authors declare no conflicts of interest.

## Data Availability

Data supporting the findings are available from the authors upon request.
